# Reciprocal regulation of HIF-1α and Uroplakin 1A promotes glycolysis and proliferation in Hepatocellular Carcinoma

**DOI:** 10.7150/jca.48132

**Published:** 2020-09-25

**Authors:** Yang Song, Hui Wang, Xue-Jing Zou, Ya-Xuan Zhang, Ze-Qin Guo, Li Liu, De-Hua Wu, Dong-Yan Zhang

**Affiliations:** 1 Department of Radiation Oncology, Nanfang Hospital, Southern Medical University, Guangzhou, GuangDong Province, 510515, China.; 2 Hepatology Unit and Department of Infectious Diseases, Nanfang Hospital, Southern Medical University, Guangzhou, GuangDong Province, 510515, China.

**Keywords:** UPK1A, HIF-1α, glycolysis, proliferation, Hepatocellular carcinoma

## Abstract

Uroplakin 1A (UPK1A) has recently been found dysregulation in many cancers. However, the functions of UPK1A and its underlying mechanisms in hepatocellular carcinoma (HCC) remain poorly understand. In the present study, we found that UPK1A was highly expressed in HCC tumor tissues compared with adjacent non-tumor tissues. Datasets from the Cancer Genome Atlas project (TCGA) and Gene expression Omnibus confirmed that UPK1A was highly expressed in HCC. High expression of UPK1A predicted poor overall survival (OS) in patients with HCC. Univariate and multivariate analysis showed that UPK1A was a significant and independent prognostic predictor for OS of patients with HCC. Functionally, silencing UPK1A suppressed HCC cell glycolysis and proliferation. Mechanistically, hypoxia-inducible factor 1α (HIF-1α) directly bound to the hypoxia response elements (HRE) of UPK1A promoter region, which led to the up-regulation of UPK1A under hypoxia. Furthermore, downregulation of UPK1A reduced key enzyme of glycolysis via regulating HIF-1α. Taken together, these data indicates the existence of a positive feedback loop between HIF-1α and UPK1A that modulates glycolysis and proliferation under hypoxia in HCC cells.

## Introduction

Reprograming of cellular metabolism to satisfy the demands of rapid proliferation, survival, invasion and metastasis is a common feature of cancer 1. This is mainly marked by the Warburg effect, which is characterized by hyper glucose metabolism 2. Genes encoding glycolytic enzymes are reported to be responsible for the Warburg effect 3. Transcription factors like hypoxia-inducible factor 1α (HIF-1α) play direct role in regulation of the Warburg effect 4. HIF-1α directly regulates a majority of glycolytic genes via binding to the hypoxia-response elements (HRE) of glycolytic gene promoter 5. HIF-1α can also indirectly regulates the Warburg effect by binding the HRE of non-glucose metabolizing enzyme gene 2, 3. However, only a small part of non-glucose metabolizing enzyme gene which can modulate the Warburg effect directly targeted by HIF-1α has been founded so far.

Uroplakin 1A (UPK1A), a highly specialized asymmetric unit membrane (AUM) characteristic of mammalian urothelial surface cells is closely related to urothelial cell differentiation 6. It has been reported that UPK1A is highly specific to normal urothelium tissues. Recently, UPK1A was found to be observed in normal genitourinary tract, prostate and uterus 7. Additionally, the expression of uroplakins markedly decreased and even totally disappeared in metastasis cancer during carcinogenesis 8, 9. However, the potential role of UPK1A in cancer development and progression remains quite contradictory. UPK1A which is significantly down-regulated in esophageal cancer acts as a tumor suppressor gene via inhibiting nuclear translocation of β-catenin and its transcriptional activity 6. UPK1A was also found to be down-regulated in colorectal cancer and gastric cancer 10. On the contrary, UPK1A was highly expressed in urinary bladder transitional cell carcinoma, and downregulation of UPK1A could suppress proliferation and enhance apoptosis of bladder transitional cell carcinoma cells 11, 12. However, the expression and functions of UPK1A in HCC remains poorly understood.

Hepatocellular carcinoma (HCC) ranks the sixth most common cancer, and accounts for the fourth leading cause of cancer related mortality worldwide 13. Adaption of glucose metabolism to satisfy the demands of rapid proliferation is commonly observed in HCC 14. The augmented glucose uptake and metabolism is generally association with poor prognosis of HCC 15. HIF-1α, a main transcription factor which regulates the Warburg effect, is highly expressed in HCC 16. Although HIF-1α has been reported to regulate glucose metabolism in HCC, the mechanism involves HIF-1α- mediated UPK1A upregulation in controlling glucose metabolism in HCC has never been discussed to our knowledge.

In the present, we report a progressively increased expression of UPK1A in HCC. Furthermore, our results provide a new mechanistic insight into the existence of a positive feedback loop between HIF-1α and UPK1A that modulates glycolysis and proliferation under hypoxia in HCC cells.

## Materials and Methods

### Cell culture

Human HCC cell line SK-Hep-1 and MHCC-97H, hepatocyte cell line MIHA, and non-small cell lung cancer cell line A549 were purchased from Cell Bank of Type Culture Collection (CBTCC, Chinese Academy of Science, Shanghai, China). Cells were cultured in Dubecco's modified Eagle's medium (DMEM, Gibco, Geand Island, NY, USA) containing 10% fetal bovine serum (Gibco) and incubated in humidified incubator. 1% O_2_ was generated by flushing a 94% N_2_/5% CO_2_ mixture into the incubator.

### Patients and Specimens

The currently study was reviewed and approved by the ethics committee of Nanfang Hospital, Southern Medical University. All patients were given informed consent prior to the inclusion of the clinical samples. Fresh HCC tissues and non-tumor samples collected from 17 HCC patients who underwent hepatectomies in Nanfang Hospital, were placed in liquid nitrogen after liver cancer resection and stored in -80°C before total RNA extraction. Formalin-fixed, paraffin-embedded HCC samples collected from 10 HCC patients were obtained from the same hospital. Patients with no evidence of extrahepatic metastasis and Child-Pugh class A of liver function at the initial stage were enrolled into the current study. All patients did not receive any treatment prior to surgery. The detailed clinical parameters of enrolled patients were listed in Table S1.

### Expression datasets

We analyzed the Cancer Genome Atlas project (TCGA dataset, https://tcga-data.nci.nih.gov/tcga/), Gene expression Omnibus (GEO, accession number GSE22058 and GSE36376), and ICGC Data Portal (ICGC-LIRI-JP, https://dcc.icgc.org/projects) datasets to explore the expression of UPK1A in HCC. The detailed clinical parameters of enrolled patients in TCGA and ICGC-LIRI-JP datasets were listed in Table S2-3. We also analyzed the correlation between expression of UPK1A and overall survival of HCC patients using data from ICGC-LIRI-JP. Kaplan Meier Plotter (http://kmplot.com/analysis/index.php?p=service) was used to analyze the correlation between the expression of UPK1A and overall survival of HCC patients. cBioPortal online software (https://www.cbioportal.org/) was used to explore the alteration frequency of UPK1A gene.

### RNA Extraction, Synthesis of cDNA and Quantitative Real-Time PCR (qRT-PCR)

These methods were described previously 17. The primers used in the present study were listed in Table S4.

### Immunohistochemistry (IHC)

IHC staining was performed to examine the expression of UPK1A in formalin, paraffin embedded HCC samples using Dako Envision System (Dako, Carpinteria, CA, USA) following the manufacturer's protocol. After baking at 65°C for 2 hours, sections were deparaffinized and rehydrated. Sections were submerged in EDTA (pH 8.0) for antigen retrieval. After incubation with 0.3% H_2_O_2_ for 15 min to block the endogenous peroxidase, the sections were incubated with antibody for UPK1A overnight at 4°C. Sections were incubated with peroxidase labeled polymer conjugated to a secondary antibody at room temperature for 50 minutes after washing. Finally, diaminobenzidine (DAB) was used for color reactions. The staining intensity was scored as follows: 0 (negative), 1 (weak), 2 (medium), 3 (strong). The score of staining extent was as follows: 0 (10%), 1 (1%-25%), 2 (26%-50%), 3 (51%-75%), and 4 (76%-100%). The total score for UPK1A was calculated based on the intensity and extent scores, ranging from 0 to 7. A total score ≥4 was defined as UPK1A high-expression.

### Gene Set Enrichment Analysis (GSEA)

This method was described previously 18.

### Small interfering RNAs (siRNA) transfection

Specific siRNAs targeting UPK1A and HIF-1α were synthesized by Genepharma Company (Jiangsu, China). The siRNAs were introduced into HCC cells using Lipofectamine RNAiMAX Transfection Reagent (Invitrogen, Carlsbad, CA, USA) according to the manufacturer's introduction. All target sequences were listed in Table S5.

### Western blot

RIPA buffer (Beyotime Biotechnology, Shanghai, China) containing Phosphatase inhibitor cocktail A (Beyotime Biotechnology) were used to extract total protein from HCC cells. The protein lysates were then separated on a 10-12% SDS-polyacrylamide gel, and transferred onto polyvinylidene fluoride (Millipore, Bedford, MA, USA) membranes. The membranes were incubated with primary antibody overnight at 4°C after blocking with 5% skimmed milk for 50 min at room temperature. The membranes were incubated with secondary antibody conjugated to horseradish peroxidase after washing with 5% Tris-Buffered Saline Tween-20 (TBST). ECL kit (Millipore, Bedford, MA, USA) was used to detect the membrane signals. The primary antibodies were listed in Table S6.

### Immunofluorescence analysis

HCC cells, cultured on coverslips, were washed twice with phosphate-buffered saline (PBS), and fixed with 4% paraformaldehyde at room temperature for 15 min. After a 10-min incubation at room temperature in 0.25% Triton X-100, the cells were blocked with 5% bovine serum albumin for 1 h at room temperature, followed by incubation overnight at 4°C with primary antibodies against UPK1A (1:100; Abcam) in PBS followed by rhodamine-conjugated goat anti-rabbit IgG (ZSCB-BIO, Beijing, China) for 1 h. The coverslips were mounted onto slides with mounting medium containing 0.2 μg/ml DAPI.

### Chromatin immunoprecipitation (ChIP) assay

ChIP experiment was performed following the protocol of ChIP assay Kit (Pierce, Rockford, IL, USA). ChIP reactions were performed with 5 μg antibody against HIF-1α or with IgG used as a negative control. Purified DNA was suspended for the following PCR assays using primers of UPK1A and LDHA promoter regions. PCR product was run on 2% agarose gels and visualized with ethidium bromide.

### Glucose consumption and lactate production

HCC cells were cultured for 48 h after treatment and the culture medium was then harvested for measurement of lactate or glucose concentration. Lactate and glucose levels were quantified using the Lactate Assay kit (Nanjing Jiancheng Bioengineering Institute, Nanjing, China) and Glucose Assay kit (Nanjing Jiancheng Bioengineering Institute) according to the manufacturer' s recommendation, respectively. Values were normalized to total protein levels determined using the Bicinchoninic Acid Protein Assay kit (Thermo Fisher Scientific, Waltham, MA, USA).

### 5-ethynyl-20-deoxyuridine (EdU) incorporation assay

Proliferating HCC cells were detected using the Cell-Light EdU Apollo 567 *in vitro* Imaging kit (RiboBio, Guangzhou, China) according to the manufacturer's protocol. The number of EdU-positive cells was counted under a confocal microscope in five random fields. Three independent experiments were performed.

### Statistical Analysis

The statistical analyses were carried out using SPSS statistical software package, version 22.0 (Abbott Laboratories, North Chicago, IL, USA). Data was shown as mean ± standard error of the mean (SEM) from three independent experiments. Survival related to UPK1A expression was evaluated using Kaplan-Meier survival analysis and log-rank test. A two-tailed *t* test was used to compare the variables of two groups. Significant differences were reflected with *P < 0.05, **P < 0.01, ***P < 0.001.

## Results

### Dysregulation of UPK1A in various cancers

**Figure 1:**
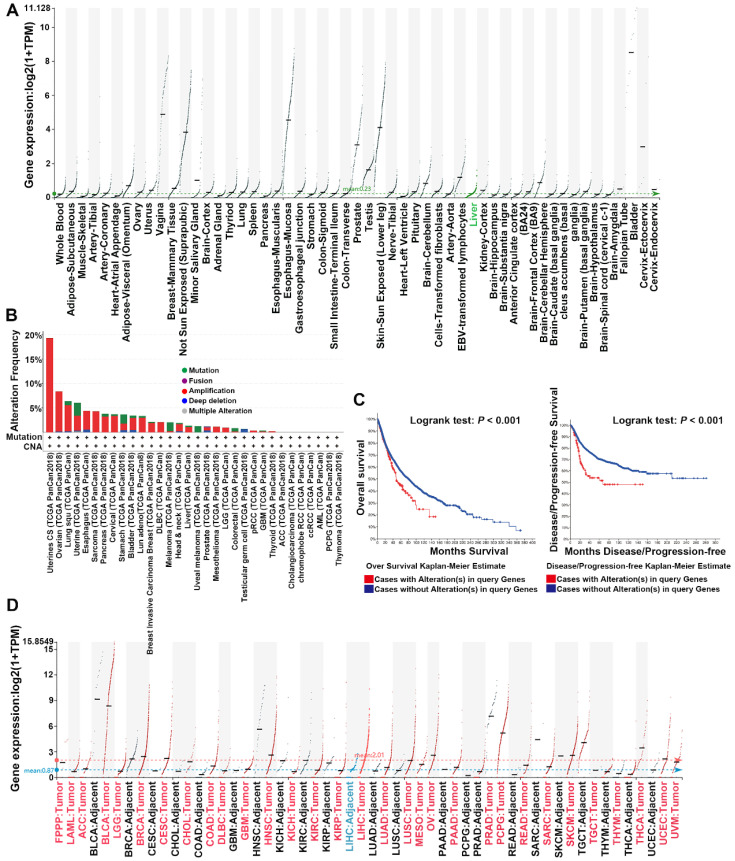
** Dysregulation of UPK1A in various cancers.** (A) Expression of UPK1A among the GTEx normal tissue RNA-seq dataset, spanning a myriad of different normal tissue types. (B) Alteration frequency of UPK1A gene was analyzed using cBioPortal online software. (C) Overall Survival and disease-free rate of patients with alterations in UPK1A gene was analyzed by cBioPortal online software. (D) Expression of UPK1A in various cancers from TCGA datasets.

UPK1A was highly expressed in genitourinary tract, uterus, testis and prostate, but barely expressed in other organs and tissues according to the Genotype-Tissue Expression (GTEx) benign tissue RNA-seq dataset (Figure 1A). However, genomic mutations especially amplification of UPK1A gene was usually observed in various cancers, including uterine carcinosarcoma, Ovarian serous cystadenocarcinoma, lung cancer, pancreatic adenocarcinoma and HCC (Figure 1B). Moreover, patients with alterations in UPK1A gene showed poorer OS and Disease-free survival than those without alterations in UPK1A gene (Figure 1C). Consistently, cancers with high UPK1A gene amplification frequency exhibited high expression of UPK1A, including Lung squamous cell carcinoma, Lung adenocarcinoma, Uterine Corpus Endometrial Carcinoma, pancreatic adenocarcinoma and HCC (Figure 1D).

### UPK1A is highly expressed in HCC

**Figure 2:**
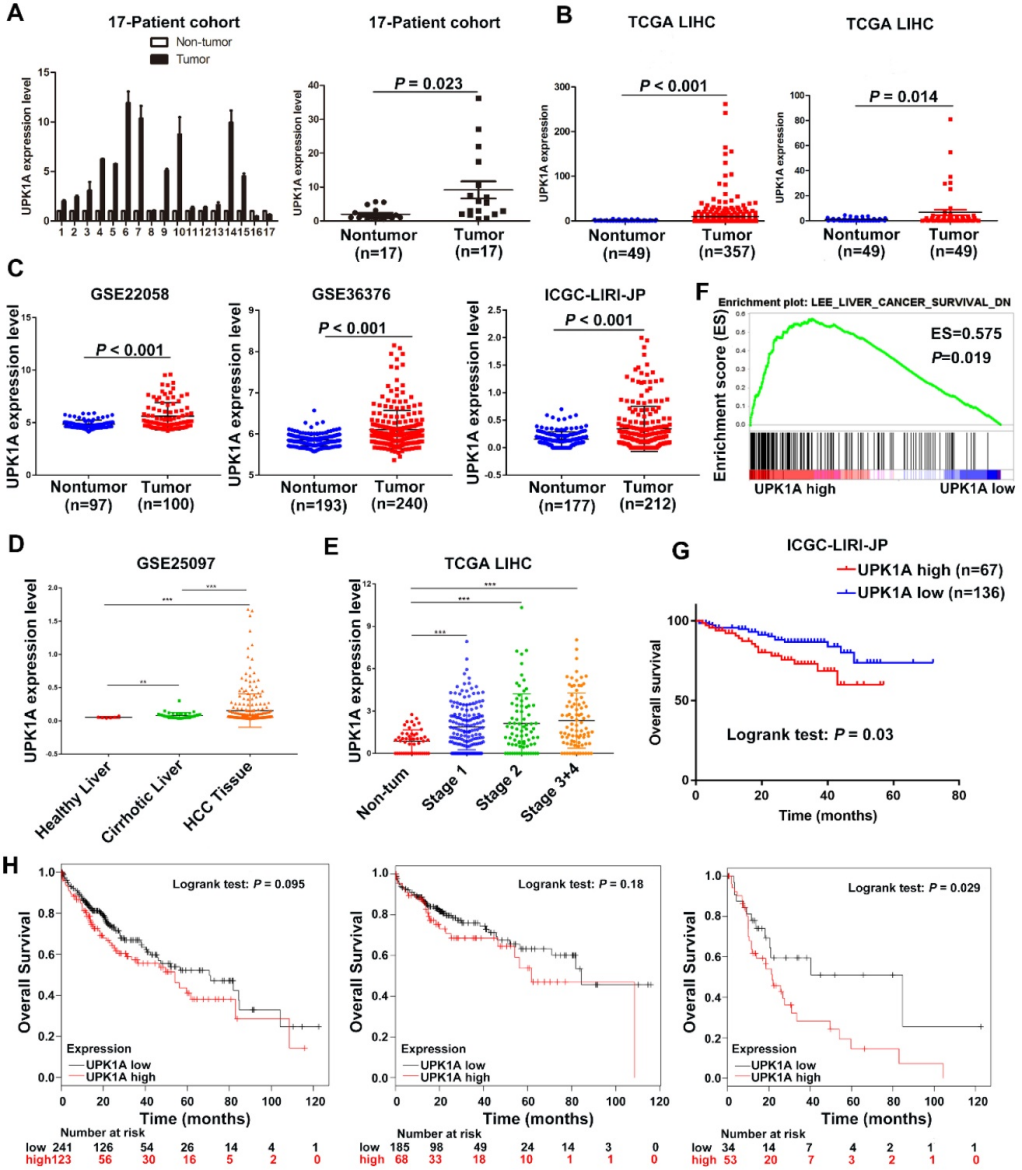
** High expression of UPK1A predicted poor clinical outcome of patients with HCC.** (A) Expression of UPK1A was evaluated in 17-Patient cohort by qRT-PCR. (B) Left panel, analysis of UPK1A expression in HCC patients from TCGA datasets (n=358). Right panel, Re-analysis of UPK1A expression level in 49 paired HCC samples and adjacent non-tumor samples from TCGA datasets (n=49). (C) Expression of UPK1A in HCC patients from GSE22058, GSE36376, and ICGC-LIRI-JP. (D) UPK1A expression level in healthy liver, cirrhotic liver, and HCC tissues. (E) UPK1A expression level in non-tumor tissue and different stage of HCC. (F) GSEA analysis suggested high UPK1A expression was association with gene signatures of poor liver cancer survival. (G) Kaplan-Meier survival analysis of overall survival of HCC patients from ICGC-LIRI-JP database. (H) Kaplan-Meier survival analysis of overall survival of HCC patients from TCGA datasets using Kaplan Meier Plotter online program.

To explore the expression of UPK1A in HCC, we examined UPK1A expression in 17 paired fresh HCC tissues and adjacent non-tumor tissues (17-Patient cohort) using qRT-PCR. As shown in Figure 2A, UPK1A was highly expressed in HCC tissues compared with adjacent non-tumor tissues. To further validate the expression level of UPK1A in HCC, we analyzed RNA-seq data of 358 HCC patients from TCGA. Similarly, the results showed that the expression level of UPK1A was significantly increased in HCC (Figure 2B, left panel, *P* < 0.001). A similar result was found when UPK1A expression was re-assessed in 50 paired HCC tissues and adjacent non-tumor samples (Figure 2B, right panel, *P* = 0.014). Consistently, data from GSE22058, GSE36376 and ICGC-LIPI-JP further confirmed that UPK1A was significantly up-regulated in HCC (Figure 2C, *P* < 0.001). Notably, data from GSE25097 and TCGA suggested that UPK1A was closely related to the development and progression of HCC (Figure 2D-E).

Next, Hematoxylin and eosin staining was performed to further distinguish HCC samples from non-tumor samples. As shown in Figure S1, UPK1A was barely expressed in adjacent non-tumor liver sample. On the contrary, UPK1A was strongly expressed in HCC tissue. Collectively, these data indicate UPK1A is highly expressed in HCC.

### High expression of UPK1A predicted poor clinical outcome of patients with HCC

A comparative microarray analysis of gene expression was carried out in the UPK1A-high and UPK1A-low expression groups by GSEA. The results showed that a high expression level of UPK1A was association with gene signatures of poor liver cancer survival (Figure 2F), which suggested that high expression of UPK1A correlated with poor prognosis in patients with HCC. Kaplan-Meier and log-rank test analyses were conducted to investigate an association between UPK1A expression level and OS for patients with HCC. The results from the ICGC-LIRI-JP datasets showed that patients with UPK1A overexpression exhibited a significantly shorter OS time as compared with those with low UPK1A expression level (Figure 1G, *P* = 0.03). Consistently, data from TCGA dataset revealed that patients with high expression of UPK1A had poorer overall survival time than those with low UPK1A expression, though the difference did not reach statistical significance. Since UPK1A closely correlated with HCC progression, we reanalyzed the data from subgroups. Interestingly, patients with high expression of UPK1A presented poorer overall survival time than those with low UPK1A expression in advanced HCC (Figure 1H). Additionally, multivariate analysis from ICGC-LIRI-JP datasets revealed that UPK1A overexpression (95% CI: 1.243-4.943, *P* = 0.01) was an independent prognostics factor for OS in HCC (Table 1 and Table 2).

### UPK1A is a direct transcriptional target of HIF-1α in HCC cells

**Figure 3:**
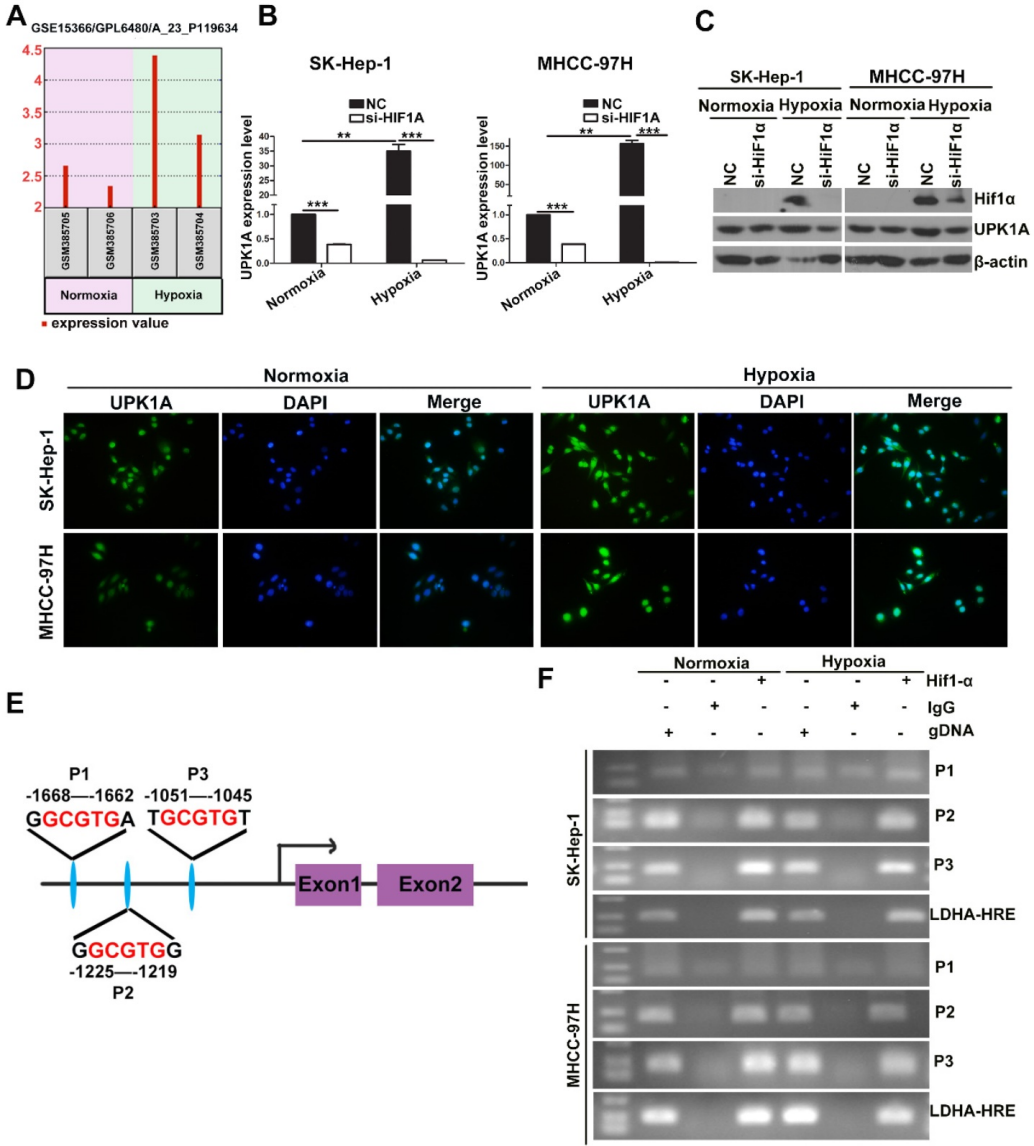
** UPK1A is a direct transcriptional target of HIF-1α.** (A) UPK1A expression levels under normoxic or Hypoxic conditions were analyzed from GSE15366. (B) mRNA expression level of UPK1A was analyzed after down-regulation of HIF-1α in SK-Hep-1 and MHCC-97H by qRT-PCR. (C) UPK1A expression level was analyzed after down-regulation of HIF-1α in SK-Hep-1 and MHCC-97H by western blot. (D) UPK1A expression level under normoxic and hypoxic conditions was analyzed in SK-Hep-1 and MHCC-97H using immunofluorescence. (E) Schematic illustration of consensus HIF-1α responsive element in UPK1A promoter region. (F) The binding of HIF-1α and the chromatin fragments corresponding to the three HREs in the promoter regions of UPK1A was verified using ChIP assay.

It has been reported that severe hypoxia condition does exist in solid tumor including HCC 19. During the development of HCC, there is an important contribution of hypoxia on prognosis via abnormal regulation of gene expression 20. To explore whether UPK1A was aberrantly expressed under hypoxic condition, we analyzed the differences between gene expression profiles (GSE15366) from Human hepatoblastoma cells HepG2 cultured under either normoxic (20% O_2_) or hypoxic (2% O_2_) conditions. Interestingly, UPK1A was highly induced under hypoxic condition (Figure 3A). HIF-1α is a key mediator of hypoxic response element. We knocked down the expression of HIF-1α in HCC cells using small interference RNAs and detected UPK1A expression level to evaluate if UPK1A was regulated by HIF-1α. As shown in Figure 3B-D, UPK1A was highly expressed under hypoxic condition. Notably, silencing HIF-1α significantly reduced the UPK1A expression either under normoxic or hypoxic conditions. We also knocked down the expression of HIF-1α in hepatocyte cell line MIHA and non-small cell lung cancer cell line A549 (Figure S2A). The expression of UPK1A remained unchanged after HIF-1α downregulation in MIHA and A549 cells (Figure S2B), indicating that hypoxic-induced upregulation of UPK1A might be tissue- and disease-specific. HIF-1α can transcriptionally regulate a vast variety of genes by directly binding to HREs within their promoters. To explore whether HIF-1α regulates UPK1A expression at the transcriptional level, we searched for the potential HREs in the promoter regions of UPK1A. Three potential HREs was found within the promoter regions of UPK1A (Figure 3E). The subsequent ChIP assays verified the binding of HIF-1α and the chromatin fragments corresponding to the three HREs in the promoter regions of UPK1A (Figure 3F). The results above demonstrate that HIF-1α transcriptionally regulates UPK1A under both normoxic and hypoxic conditions.

### UPK1A is essential for hypoxia-enhanced glycolysis and proliferation in HCC cells

**Figure 4:**
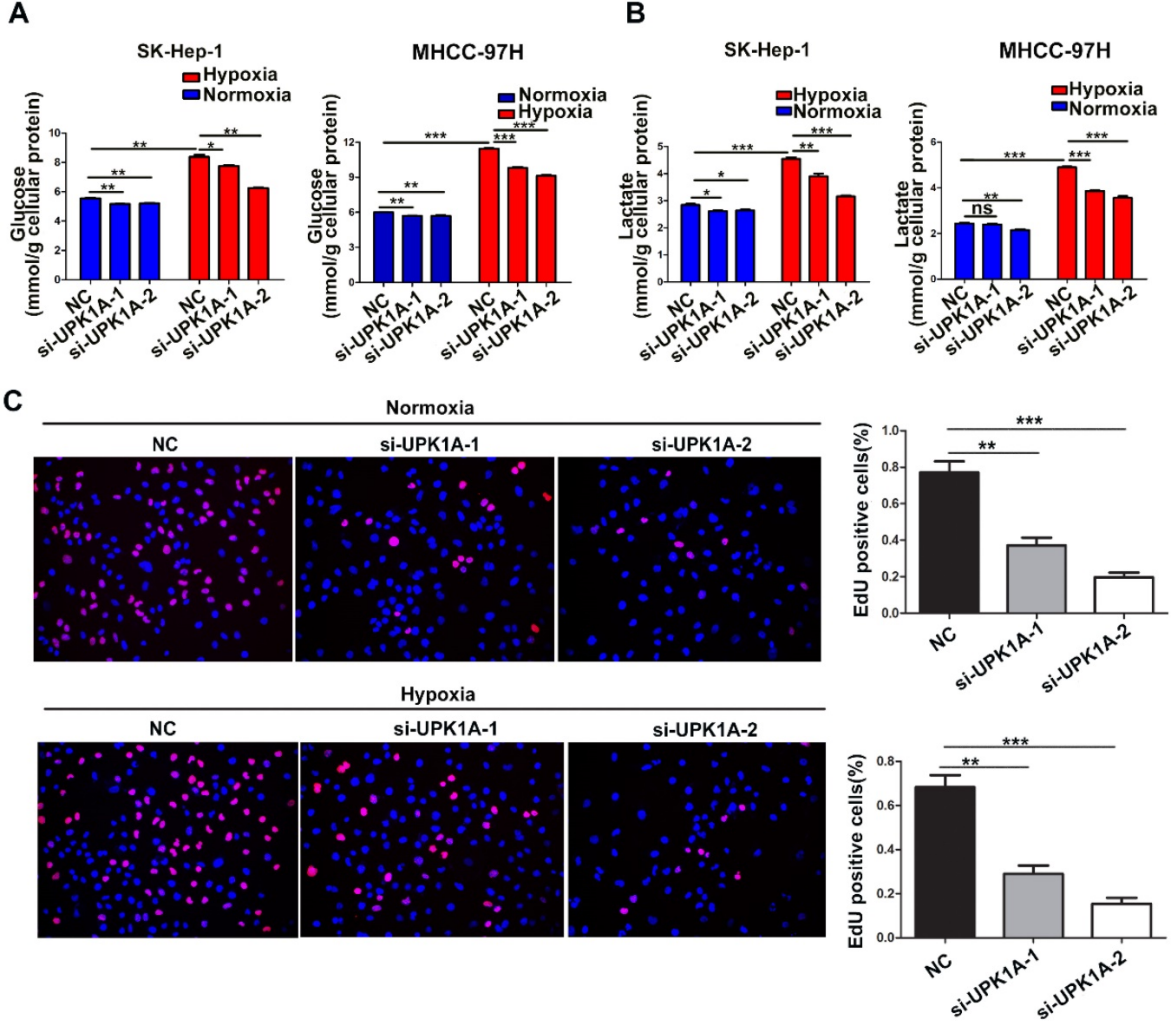
** UPK1A is essential for hypoxia-enhanced glycolysis and proliferation.** (A) Glucose consumption(A) and lactate production (B) was analyzed after down-regulation of UPK1A in SK-Hep-1 and MHCC-97H cell. (C) Effect of UPK1A down-regulation on SK-Hep-1 cell proliferation was evaluated by EdU assay.

**Figure 5:**
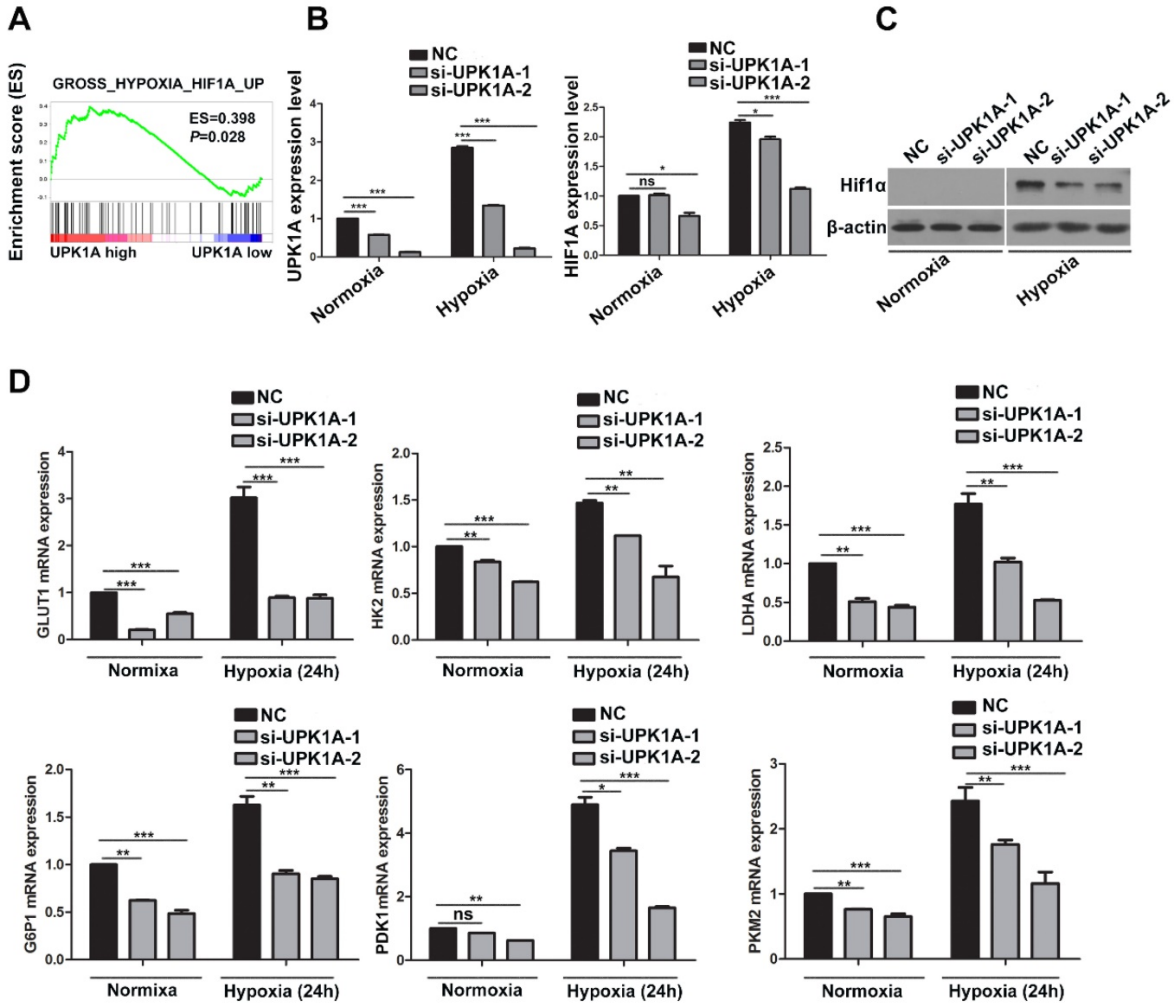
** Downregulation of UPK1A inhibits HCC cell glycolysis via regulating HIF-1α signaling.** (A) GSEA analysis indicated that UPK1A positively correlated with gene signature involved in hypoxia and HIF-1α signaling. (B) mRNA expression level of UPK1A and HIF-1α after silencing UPK1A in SK-Hep-1 cell were detected using qRT-PCR. (C) HIF-1α expression level after silencing UPK1A in SK-Hep-1 cell was evaluated using western blot. (D) Expression level of HIF-1α target genes after silencing UPK1A in SK-Hep-1 cell were detected using qRT-PCR.

To investigate the functional role of hypoxia-induced UPK1A, we used 2 different UPK1A-targeting siRNAs to knock down UPK1A expression. The knockdown efficiency of UPK1A was examined using qRT-PCR. The results showed that the two siRNAs successfully silenced UPK1A expression (Figure 5B). It has been well known that hypoxic condition causes increased glycolysis in HCC cells 21. Consistent with this, hypoxia treatment led to a significantly increase in glucose consumption and lactate production (Figure 4A-B). Notably, all these hypoxia-induced effects were strongly reversed by UPK1A knockdown (Figure 4A-B). However, downregulation of UPK1A had no or little impact on the glucose consumption in MIHA and A549 cells (Figure S2C). Given that glycolysis contributes to HCC progression, we examined the biological function of UPK1A in HCC. Silencing UPK1A obviously inhibited HCC cell proliferation both in normoxic and hypoxic conditions (Figure 4C).

### Downregulation of UPK1A inhibits HCC cell glycolysis via regulating HIF-1α signaling

We next performed GSEA analysis on tumor samples from TCGA dataset to explore the mechanisms underlying UPK1A involved in HCC glycolysis and proliferation. The results demonstrated that high expression of UPK1A positively correlated with gene signature involved in hypoxia and HIF-1α signaling (Figure 5A). We thus assumed that UPK1A might regulate HIF-1α or its downstream targets. To test our hypothesis, we detected the expression of HIF-1α using qRT-PCR and western blot. The results revealed that downregulation of UPK1A remarkably reduced the expression of HIF-1α in both mRNA and protein level (Figure 5B-C), which suggested that UPK1A might regulate HIF-1α at transcriptional level in HCC cells. However, downregulation of UPK1A did not alter the expression of HIF-1α in MIHA and A549 cells (Figure S 2D). HIF-1α transactivates hypoxia-inducible glycolysis enzymes by binding to the HREs in promoters of these genes. Our results revealed that downregulation of UPK1A significantly decreased the expression of HIF-1α targets genes involved in glycolysis including GLUT1, HK2, LDHA G6PI, PDK1, and PKM2 (Figure 5D). Taken together, our results suggest that downregulation of UPK1A inhibits HCC cell glycolysis via regulating HIF-1α signaling.

## Discussion

UPK1A, an integral protein which belongs to the transmembrane 4 superfamily (TM4SF), is thought to be specific to normal urothelium 22, 23. Some of the TM4SF superfamily members have been reported to play crucial roles in the regulation of cell proliferation, differentiation, motility and invasion 24, the physiological and biological function of UPK1A remains largely unrevealed. In the present study, we found that down-regulation of UPK1A significantly reduced HCC cell proliferation and glycolysis. Further investigation revealed UPK1A inhibits HCC cell proliferation and glycolysis via regulating HIF-1α signaling.

It has been reported that UPK1A was dysregulated in various cancer. However, the potential role of UPK1A in cancer development and progression remains quite contradictory. UPK1A was down-regulated and identified as a tumor suppressor in esophageal squamous cell carcinoma 6, colorectal cancer 25, and gastric cancer 10. UPK1A was found to be up-regulated in urinary bladder transitional cell carcinoma instead 12. There seemed to be more complex roles for UPK1A regarding development and progression in different cancer types. In esophageal squamous cell carcinoma, hypermethylation in the promoter region of UPK1A accounts for the inactivation and down-regulation of UPK1A 6. The mechanisms underlying the up-regulation of UPK1A in cancer like urinary bladder transitional cell carcinoma is largely unknown. Through genomic analysis we found that genomic mutations especially amplification of UPK1A gene was usually observed in various cancers, including uterine carcinosarcoma, ovarian serous cystadenocarcinoma, lung cancer, pancreatic adenocarcinoma. Consistently, cancers with high UPK1A gene amplification frequency exhibited high expression of UPK1A in lung squamous cell carcinoma, lung adenocarcinoma, uterine corpus endometrial carcinoma, pancreatic adenocarcinoma, which indicates UPK1A gene amplification may account for upregulation of UPK1A in these cancers. Interestingly, patients with alterations in UPK1A gene showed poorer OS and Disease-free survival than those without alterations in UPK1A gene. Taken together, dysregulations of UPK1A may play crucial roles in cancers.

Hypoxia, commonly seen in solid tumor, is considered to play crucial roles in HCC progression and development 20. Cancers cells inside solid tumor suffer from hypoxia due to abnormal growth of tumor, rapidly increased tumor size, abnormal growth of tumor vasculature, and so on 26. Cancer cells prefer to glycolysis instead of oxidative phosphorylation under hypoxic condition 27. HIF-1α, a major transcription factor which regulates a majority of glycolytic genes via binding to the HRE of glycolytic gene promoter, is highly expressed in HCC and predicts poor prognosis for HCC patients 28-30. Here, we found HIF-1α directly bound to the HREs of UPK1A promoter region by ChIP assay, leading to the upregulation of UPK1A under hypoxic condition, which might account for the upregulation of UPK1A in HCC. GSEA analysis form TCGA dataset revealed that upregulation of UPK1A correlated with HIF-1α signaling pathway, which inspired us to determine whether UPK1A regulated HIF-1α signaling. Notably, down-regulation of UPK1A significantly decreased mRNA and protein expression level of HIF-1α, suggesting UPK1A regulated HIF-1α at transcriptional level. Consistently, UPK1A silencing significantly decreased the expression of HIF-1α targets genes involved in glycolysis including GLUT1, HK2, LDHA G6PI, PDK1, and PKM2. These results indicate that HIF-1α upregulates UPK1A which in turn increases HIF-1α and its downstream targets to promotes HCC cell glycolysis and proliferation under hypoxia.

Despite advance treatment strategies have been improved, the survival rate remains unsatisfactory for patients with HCC 31. Therefore, novel risk markers to guide disease managements of HCC patients for the improvement of their survival are urgently needed. Upregulation of UPK1A in HCC patients was significantly related with poor prognosis by Kaplan-Meier survival analysis, suggesting that UPK1A may become a novel marker to predict OS for HCC patients. Univariable and multivariable Cox proportional hazard regression analysis show that upregulation of UPK1A was an independent factor in predicting OS for patients with HCC. In conclusion, our results indicate that UPK1A, significantly upregulated in HCC, predicts poor prognosis for patients with HCC. In addition, we provide a new mechanistic insight into the existence of a positive feedback loop between HIF-1α and UPK1A that modulates glycolysis and proliferation under hypoxia in HCC cells.

## Supplementary Material

Supplementary figures and tables.

## Figures and Tables

**Table 1:** The associations of UPK1A expression with clinicopathological characteristics in ICGC-LIRI-JP (n=203) cohort

Feature	Low expression of UPK1A (n=136)	High expression of UPK1A (n=67)
**Age (years)**		
≥50	127	61
<50	9	6
**Gender**		
Male	106	47
Female	30	20
**Virus**		
HBV	30	23
HCV	82	35
HBV and HCV	2	2
Negative	22	7
**TNM stage**		
I+II	91	38
III+IV	45	29
**Portal vein invasion**		
Positive	29	20
Negative	107	47
**Vein invasion**		
Positive	48	17
Negative	88	50
**Bile duct invasion**		
Positive	47	7
Negative	89	60
**Fibrosis**		
Yes	130	66
No	6	1
**Alcohol**		
Yes	90	43
No	40	24
NA	6	0
**Smoking**		
Yes	89	43
No	42	23
NA	5	1

Abbreviations: NA, not available.

**Table 2:** Univariate and multivariate analyses of OS in ICGC-LIRI-JP (n=203) cohort by Cox regression analysis

Variables	Univariate analysis			Multivariate analysis		
	HR	CI (95%)	*P* value	HR	CI (95%)	*P* value
Age (years)	0.922	0.325-2.616	0.878			
Gender	0.536	0.265-1.083	0.082			
Virus (HBV or/and HCV)	1.654	0.506-5.408	0.406			
TNM stage	2.830	1.446-5.540	0.002*			
Portal vein invasion	3.135	1.595-6.160	0.001*			
Vein invasion	2.394	1.172-4.891	0.017*			
Fibrosis	1.158	0.276-4.853	0.841			
Alcohol	0.476	0.242-0.936	0.031*	0.311	0.150-0.646	0.002*
Smoking	0.758	0.391-1.471	0.413			
UPK1A	2.121	1.092-4.119	0.026*	2.479	1.243-4.943	0.010*

Abbreviations: HBV, hepatitis B virus; HCV, hepatitis C virus; CI, confidence interval; HR, hazard radio; *The values had statistically significant differences.

## Abbreviations

AUM: asymmetric unit membrane
CBTCC: Cell Bank of Type Culture Collection
ChIP: Chromatin immunoprecipitation
DMEM: Dubecco's modified Eagle's medium
DAB: diaminobenzidine
EdU: 5-ethynyl-20-deoxyuridine
GEO: Gene expression Omnibus
GSEA: Gene Set Enrichment Analysis
GTEx: Genotype-Tissue Expression
HCC: hepatocellular carcinoma
HIF-1α: hypoxia-inducible factor 1α
HRE: hypoxia response elements
HC: immunohistochemistry
OS: overall survival
PBS: phosphate-buffered saline
qRT-PCR: Quantitative Real-Time PCR
siRNA: Small interfering RNAs
SEM: mean ± standard error of the mean
TBST: Tris-Buffered Saline Tween-20
TCGA: The Cancer Genome Atlas project
TM4SF: transmembrane 4 superfamily
UPK1A: Uroplakin 1A
