# Supplementary material for: Reciprocal regulation of HIF-1α and Uroplakin 1A promotes glycolysis and proliferation in Hepatocellular Carcinoma
Source: J Cancer. 2020 Sep 25;11(22):6737–47. doi: 10.7150/jca.48132 (PMC7545691; doi:10.7150/jca.48132)
Supplement: Supplementary file 1 — Supplementary figures and tables. [file jcav11p6737s1.pdf]

Reciprocal Regulation of HIF-1α and Uroplakin 1A promotes glycolysis and proliferation in hepatocellular carcinoma

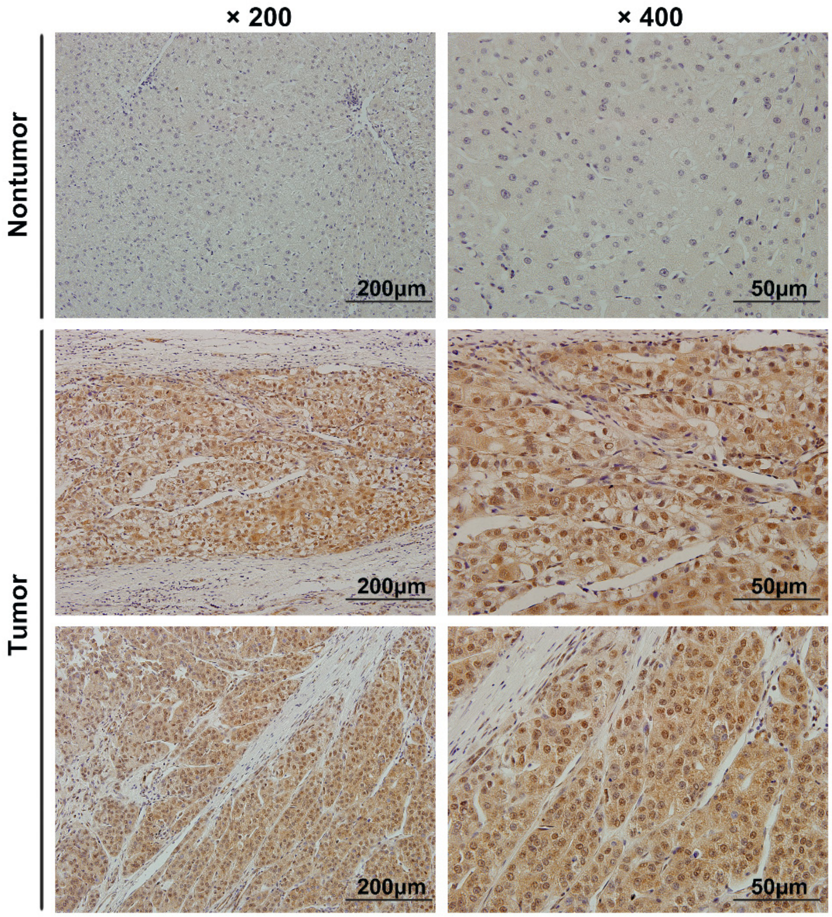

Figure S1. Representative images of UPK1A expression in non-tumor tissues and HCC tissues by IHC assay (magnification: left, ×200; right, ×400).

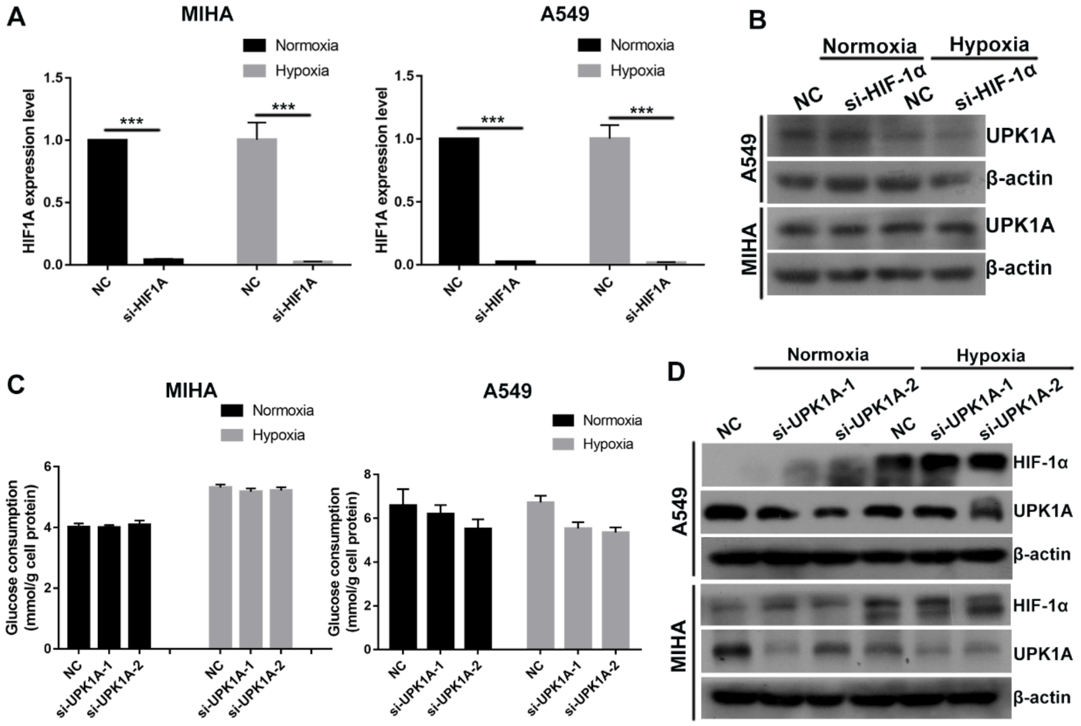

Figure S2. Effect of UPK1A downregulation on glycolysis in MIHA and A549 cells. A. Expression of HIF-1α was knocked down in MIHA and A549 cells. B. Expression of UPK1A was detected by western blot after HIF-1α downregulation in MIHA and A549 cells. C. Glucose consumption was analyzed after down-regulation of UPK1A in MIHA and A549 cells. D. HIF-1α expression level was measured by western blot after down-regulation of UPK1A in MIHA and A549 cells.

Supplementary Tables

Table S1

Clinicopathological characteristics of 17 HCC patients

Feature	N（%）
Age(years)	
≤55	10 (58.8)
>55	7 (41.2)
Gender	
Male	15 (88.2)
Female	2 (11.8)
Tumour size (cm)	
≤5	4 (23.5)
>5	13 (76.5)

Edmondson Grade	
I-II	7 (41.2)
III-IV	10 (58.5)
BCLC stage	
A	10 (58.8)
B+C+D	7 (41.2)
Liver cirrhosis	
Yes	10 (58.8)
No	7 (41.2)
Portal vein tumour thrombus	
Yes	8 (47.1)
No	9 (52.9)
No. tumour	
Solitary	14 (82.4)
Multiple	3 (11.6)
HBV	
With	13 (76.5)
Without	4 (23.5)

Table S2

Clinicopathological characteristics of 357 HCC patients from TCGA datasets

Feature	N（%）
Age(years)	
≤55	121 (33.9)
>55	236 (66.1)
Gender	
Male	241 (67.5)
Female	116 (32.5)
Race	
White	172 (48.2)
Black	16 (4.5)
Asian	158 (44.3)
other	11 (3.0)
Neoplasm histologic grade	
1-2	221 (61.9)
3-4	131 (36.7)
NA	5 (1.4)
Viral hepatitis serology	
HBV	57(16.0)
HCV	19 (5.3)
HBV+HCV	37(10.4)
NA	244 (68.3)
Pathologic Stage	
I-II	249 (69.7)
III-IV	87 (24.4)
NA	21(5.9)
Vascular invasion	
With	105 (29.4)
Without	198 (55.5)
NA	54 (15.1)
Child-Pugh grade	
A	212 (59.4)
B	20 (5.6)
NA	125 (35.0)

Abbreviations: NA, not available.

Table S3

Clinicopathological characteristics of HCC patients in ICGC-LIRI-JP (n=203) cohort

Feature	N (%)
Age(years)	
≥50	188 (92.6)
<50	15 (7.4)
Gender	
Male	153 (75.4)
Female	50 (24.6)
Virus	
HBV	53 (26.1)
HCV	117 (57.6)
HBV and HCV	4 (1.9)
Negative	29 (14.4)
TNM stage	
I+II	129 (63.5)
III+IV	74 (36.5)
Portal vein invasion	
Positive	49 (24.1)
Negative	154 (75.9)
Vein invasion	
Positive	65 (32.0)
Negative	138 (68.0)
Bile duct invasion	
Positive	54 (26.6)
Negative	149 (73.4)
Fibrosis	
Yes	196 (96.6)
No	7 (3.4)
Alcohol	
Yes	133 (65.5)
No	64 (31.5)
NA	6 (3.0)
Smoking	
Yes	132 (65.0)
No	65 (32.0)
NA	6 (3.0)

Abbreviations: NA, not available.

Table S4

Primer sequence used in this study

Gene	sequence
β-actin-F	5′ TCAAGATCATTGCTCCTCCTGA 3′

β-actin-R	5′ CTCGTCATACTCCTGCTTGCTG 3′
UPK1A-F	5′ GAGGCTGCAGACAGAGAAGG 3′
UPK1A-R	5′ TGAAGAAGGAGAAGCCGCAG 3′
HIF1A-F	5′ TCTGGATGCTGGTGATTTGG 3′
HIF1A-R	5′ GCACCAAGCAGGTCATAGGT 3′
GLUT1-F	5′ CGGGCCAAGAGTGTGCTAAA 3′
GLUT1-R	5′ TGACGATACCGGAGCCAATG 3′
HK2-F	5′ AACAGCCTGGACGAGAGCAT 3′
HK2-R	5′ GTCCACGCTTGGTGAAATCG 3′
LDHA-F	5′ ATCTTGACCTACGTGGCTTGGA 3′
LDHA-R	5′ CCATACAGGCACACTGGAATCTC 3′
PDK1-F	5′ ACCAGGACAGCCAATACAAG 3′
PDK1-R	5′ CCTCGGTCACTCATCTTCAC 3′
PKM2-F	5′ TCCGACACAGTCTCCTGGAC 3′
PKM2-R	5′ AAGCTTCCCCAGCTGTTCTC 3′
G6P1-F	5′ GCATTCCTGTGA CTGAGCAG 3′
HRE1-F	5'GTAGAGATAGGGTTTCACCATGTTAG 3'
HRE1-R	5' CACTGGTTTGGTAATAAGATACATG 3'
HRE2-F	5'CTGACCTAAGGTGATCCCCCC 3'
HRE2-R	5'CACTGAAAATCCCATCGTC 3'
HRE3-F	5'GATGATACCCATTTCGCAGATGGGC 3'
HRE3-R	5'GGAGAAGAACGTGCTAGAAAATGAG 3'
LDHA-HRE-F	5'TTGGAGGGCAGCACCTTACTTAGA 3'
LDHA-HRE-R	5'GCCTTAAGTGGAACAGCTATGCTGAC 3'

Table S5

siRNA sequence used in this study

siRNA	sense sequence	anti-sense sequence
si-UPK1A-1	5'GCGUCAUGAUUGAG CAAGATT 3'	5'UCUUGCUCAAUCAUGA CGCTT 3'
si-UPK1A-2	5'CACAUGGACUACCU GUUCATT 3'	5'UGAACAGGUAGUCC AUGUGTT 3'
si-HIF-1α	5'GGACACAGAUUUAG ACUUGTT 3'	5'CAAGUCUAAAUCUG UGUCCTT 3'

Table S6

Information on antibodies used in this study

Antibody	WB	IHC	IF	Specificity	Company

UPK1A (ab185970)	1: 1000	/	/	Rabbit polyclonal	Abcam
UPK1A (sc-366947)	/	1:100	1:100	Rabbit polyclonal	Santa Cruz Biotechnology
β-actin (sc-8432)	1:2000	/	/	Mouse monoclonal	Santa Cruz Biotechnology
HIF-1α (20960-1-AP)	1:1000	/	/	Rabbit polyclonal	Proteintech Group
